# Critical Role of Autophagy in the Processing of Adenovirus Capsid-Incorporated Cancer-Specific Antigens

**DOI:** 10.1371/journal.pone.0153814

**Published:** 2016-04-19

**Authors:** Sarah R. Klein, Hong Jiang, Mohammad B. Hossain, Xuejun Fan, Joy Gumin, Andrew Dong, Marta M. Alonso, Candelaria Gomez-Manzano, Juan Fueyo

**Affiliations:** 1 Department of Neuro-Oncology, Brain Tumor Center, The University of Texas MD Anderson Cancer Center, Houston, Texas, United States of America; 2 Cancer Biology Program, The University of Texas Graduate School of Biomedical Sciences at Houston, Houston, Texas, United States of America; 3 Department of Neurosurgery, Brain Tumor Center, The University of Texas MD Anderson Cancer Center, Houston, Texas, United States of America; 4 Department of Medical Oncology, Clínica Universidad de Navarra, University of Navarra, Pamplona, Spain; 5 Department of Genetics, Brain Tumor Center, The University of Texas MD Anderson Cancer Center, Houston, Texas, United States of America; University of Michigan School of Medicine, UNITED STATES

## Abstract

Adenoviruses are highly immunogenic and are being examined as potential vectors for immunotherapy. Infection by oncolytic adenovirus is followed by massive autophagy in cancer cells. Here, we hypothesize that autophagy regulates the processing of adenoviral proteins for antigen presentation. To test this hypothesis, we first examined the presentation of viral antigens by infected cells using an antibody cocktail of viral capsid proteins. We found that viral antigens were processed by JNK-mediated autophagy, and that autophagy was required for their presentation. Consistent with these results, splenocytes isolated from virus-immunized mice were activated by infected cells in an MHC II-dependent manner. We then hypothesize that this mechanism can be utilized to generate an efficient cancer vaccine. To this end, we constructed an oncolytic virus encompassing an EGFRvIII cancer-specific epitope in the adenoviral fiber. Infection of cancer cells with this fiber-modified adenovirus resulted in recognition of infected cancer cells by a specific anti-EGFRvIII antibody. However, inhibition of autophagy drastically decreased the capability of the specific antibody to detect the cancer-related epitope in infected cells. Our data suggest that combination of adenoviruses with autophagy inducers may enhance the processing and presentation of cancer-specific antigens incorporated into capsid proteins.

## Introduction

Oncolytic adenoviruses, such as Δ24-RGD (Delta-24-RGD),[1, 2] are highly immunogenic.[3] The current hypothesis to explain the antitumor mechanism in patients treated with oncolytic adenoviruses is that the main effect is achieved through the trigger of an antitumor immune response.[4] Autophagy is a recognized feature of cells infected with adenoviruses[5, 6] and is an important component of the lysis process.[7] Xenophagy[8] and pathogen-derived antigen processing[9, 10] are fundamental functions of autophagy in cells infected with viruses. Autophagy degrades intracellular content, processing epitopes to be loaded onto major histocompatibility complex (MHC) molecules for presentation at the surface of immune cells.[9–11] However, the way in which cancer cells process adenovirus-derived antigens is currently unknown.

The modulation of autophagy in mammalian cells is best described in cells undergoing starvation.[12–14] In addition to the canonical pathway,[12–14] the c-Jun N-terminal kinase (JNK) signal transduction pathway is activated in cells undergoing autophagy.[15] Moreover, JNK activation has been observed in cells infected with viruses.[16] Despite the clear role that JNK plays on the regulation of innate and adaptive immune responses,[17] including the capability to potentiate the immune response to viral pathogens by up-regulating the expression of proinflammatory cytokines,[18–20] the direct function of JNK in pathogen-derived antigen processing and presentation has not yet been defined.

We have recently reported that JNK expression and activation by phoshporylation is required for the induction of productive autophagy in adenovirus-infected cells.[16] In this study, we found that adenoviral structural proteins interact with autophagy cargo-receptor proteins leading to its degradation in the autophagolysosomes. Autophagy seems to be essential for the presentation adenovirus-derived antigens because inactivation of the autophagy regulator JNK or direct inactivation of autophagy drastically limited the recognition of cancer-infected cells by primed immune cells and antibodies against capsid or cancer-specific ectopic epitopes encoded by the adenovirus fiber.

## Material and Methods

### Cell culture

U87 MG glioma, HeLa cervical cancer, and A549 lung cancer cell lines were all obtained from ATCC. HeLa cells were cultured in DMEM (1X), and A549 cells were cultured in DMEM/F-12 50:50 medium (Invitrogen). U87 MG cells (ATCC) were cultured in MEM with 10% FBS and 1% essential amino acids. Wild-type and *JNK1/2-/-* mouse embryo fibroblasts (MEFs) were provided by Roger Davis (University of Massachusetts Medical School, MA). Wild-type and knock-out Atg5 MEFs (*Atg5-/-*) are a generous gift from Noboru Mizushima (Tokyo Medical and Dental University, Tokyo, Japan). Wild-type and knock-out p62 MEFs (*p62-/-)* were a generous gift from Jorge Moscat (Sanford-Burnham Medical Research Institute, La Jolla, CA). MEFs were cultured in DMEM/F12 50:50 medium. Cells were cultured with 10% FBS at 37°C in 5% CO_2_ in air.

### Chemicals and antibodies

The JNK kinase inhibitor SP600125 and bafilomycin A1 were purchased from Sigma-Aldrich, St Louis, MO, USA). Rapamycin was purchased from Calbiochem (San Diego, CA, USA). Recombinant human protein interferon gamma (IFN-γ) was purchased from Prospec (East Brusnwick, NJ, USA). Antibodies to LC3, Beclin 1, and ubiquitin were purchased from Cell Signaling Technologies (Beverly, MA, USA). Anti-p62 and anti-Actin antibodies were purchased from Santa Cruz Biotechnology (Dallas, Texas, USA). Anti-adenoviral fiber was obtained from ThermoScientific (Waltham, MA, USA). EGFRvIII was detected with a monoclonal antibody (L8A4) obtained as a generous gift by Dr. Bigner (Duke University, NC, USA). Allophycocyanin (APC)-conjugated anti-mouse secondary antibodies and fluorescein isothiocyanate (FITC)-conjugated secondary antibody were purchased from Santa Cruz Biotechnology.

### Adenoviral production and infection

Δ24RGD and AdWT adenoviruses were produced as described previously.[1] Δ24FvIII was constructed by inserting the EGFRvIII epitope (LEEKKGNYVVT)[21] into the HI loop of fiber protein between amino acids 543 and 544.[22] First, the sequence coding for the EGFRvIII epitope was incorporated into the fiber gene in vector pXK-F containing the *Xba* I–*Kpn* I fragment of pVK503C[23] via site-directed mutagenesis. Next, the Xba I-Kpn I fragment from the resulting vector pXK-FvIII was co-transduced with *Swa*I-linearized pVK500C.delta-24 into *E*. *coli* BJ5183 for homologous recombination as described previously.[24] The virus was then rescued in 293 cells and propagated in A549 cells, as previously described.[24] Adenoviruses were added to cell culture media at the indicated multiplicity of infection (MOI).

### Western blot analysis

Cells were lysed with RIPA lysis buffer. Protein concentrations were determined with use of a Bio-Rad protein assay. Protein samples (25 μg) in 1X SDS loading buffer were boiled and loaded into Tris-glycine sodium dodecyl sulfate–polyacrylamide gel electrophoresis (SDS-PAGE) gels (Invitrogen, Carlsbad, CA, USA). Samples were separated by using electrophoresis. Gels were then transferred to polyvinylidene fluoride membranes, which were blocked by using 5% non-fat milk in 1X TBS-T (0.025% Tween) for 1 h at room temperature and then incubated overnight at 4°C in a primary antibody at appropriate dilutions. Membranes were washed three times with TBS-T (0.05% Tween) and then incubated at room temperature for 1 h with appropriate secondary antibodies (Santa Cruz Biotechnology; 1:4000) prepared in 1% non-fat milk in TBS-T. SuperSignal West Femto chemiluminescent substrate (ThermoScientific) and HyBlot CL autoradiography film (Denville Scientific Inc., South Plainfield, NJ, USA) were used to visualize protein bands.

### RNA interference

Wild-type MEF cells were seeded into 6-well plates 24 h before small-interfering RNA (siRNA) transfection. INTERFERin (Polyplus Transfection (Illkirch-Graffenstaden, France) was used to insert the siRNAs into cells according to the manufacturer’s protocol. Briefly, 50 nM siRNA oligonucleotides were combined with 10 μL of transfection reagent and added to the cells after 15 min of incubation at room temperature. siRNA oligonucleotides for mouse MHC class I, MHC class II, and non-coding regions were purchased from Santa Cruz Biotechnology.

### Co-immunoprecipitation

Cells were infected with AdWT or Δ24RGD for up to 48 h. Floating and attached cells were collected and lysed with immunoprecipitation (IP) lysis buffer (100 mM NaCl, 50 mM Tris-HCl [pH 7.4], 2 mM EDTA, 10% glycerol, 1% IgePal CA-630). The protein samples were pre-cleared with protein A and protein G agarose beads. Anti-adenoviral fiber or anti-p62 antibodies were added to the lysates at a dilution of 1:100, and complexes of proteins were immobilized with protein A and protein G (1:1) agarose beads. Immunoprecipitated samples, pre-cleared beads, and 5% input were analyzed by Western blotting. IP detection secondary antibody was used to detect proteins bound by all anti-rabbit and anti-mouse primary IgGs.

### Flow cytometry

Cells were treated for respective time-points, trypsinized, and collected in phosphate-buffered saline (PBS) with 1% bovine serum albumin (BSA). Cells were treated when indicated with IFN-γ (300 units/mL) and bafilomycin A1 (Sigma-Aldrich). Live cells were stained for adenoviral antigens by using a combination of mouse anti-adenovirus (blend) coating (1:75; Millipore, Billerica, MA, USA) and mouse anti-adenovirus fiber antibodies (1:75; ThermoScientific) for 30 min at 4°C. After the cells were washed, they were stained with APC-conjugated anti-mouse secondary antibodies (1.75; Santa Cruz Biotechnology) for 30 min at 4°C. For detection of the EGFRvIII epitope (LEEKKGNYVVT), the cells were stained with a monoclonal antibody (L8A4) followed by FITC-conjugated secondary antibody staining. The live cells were analyzed with use of a BD FACSCalibur (Becton-Dickenson, Franklin Lakes, NY, USA). Dead cells were excluded by using propidium iodide or ethidium homodimer-1 staining before analysis. The data represent at least four independent experiments.

### Splenocyte activation

All animal studies were performed in the veterinary facilities at The University of Texas MD Anderson Cancer Center in accordance with institutional guidelines and the Guide for the Care and Use of Laboratory Animal. The study was approved by The University MD Anderson Cancer Center Institutional Animal Care and Use Committee (IACUC). C57BL/6 mice (10–12 weeks old) were infected intracranially with two injections (days 0 and 3) of 1 × 10^8^ pfu/mouse Δ24RGD via the guide-crew system, as described previously.[1] The animals were euthanized with use of a CO_2_ chamber; the spleens were removed andsmashed trough 100-micrometer strainer;[25] and splenocytes were washed with PBS. Red blood cells were removed via ACK lysing buffer (Lonza, Houston, TX, USA). The wild-type and JNK knock-out MEFs were seeded at 1 × 10^5^ cells in a 6-well plate (in triplicate). The cells were infected with 100 MOI of Δ24RGD for 24 h, trypsinized, and re-plated into a 96-well plate at 5 × 10^4^ cells per well in RPMI 1640 media with 10% FBS and 55 μM beta-mercaptoethanol. Splenocytes (1 × 10^6^ cells) were incubated with the MEFs in culture for 24 h. For experiments including blocking antibodies, pre-infected MEF cells were incubated with 2 μg of anti-mouse MHC class II (I-A/I-E), anti-mouse MHC class I (H-2Kd) (eBioscience, San Diego, CA), or mouse IgG (Santa Cruz) 2 h before co-culture with primed splenocytes. Splenocytes were then incubated with the pre-infected MEF cells and blocking antibodies for 24 h. A Mouse IFN-γ ELISA Kit (ThermoScientific) was used to evaluate the concentration of IFN-γ in 50 μL of media extracted from the co-culture. ELISA was performed according to the manufacturer’s instructions, and absorbance was analyzed via an Omega microplate reader (BMG Labtech, Ortenberg, Germany).

### Statistical analysis

A two-tailed Student *t*-test was used to determine the statistical significance of treated and infected samples relative to control samples. P values less than 0.05 were accepted as statistically significant.

## Results

### JNK-mediated autophagy regulates the presentation of adenovirus-derived antigens

**Fig 1:**
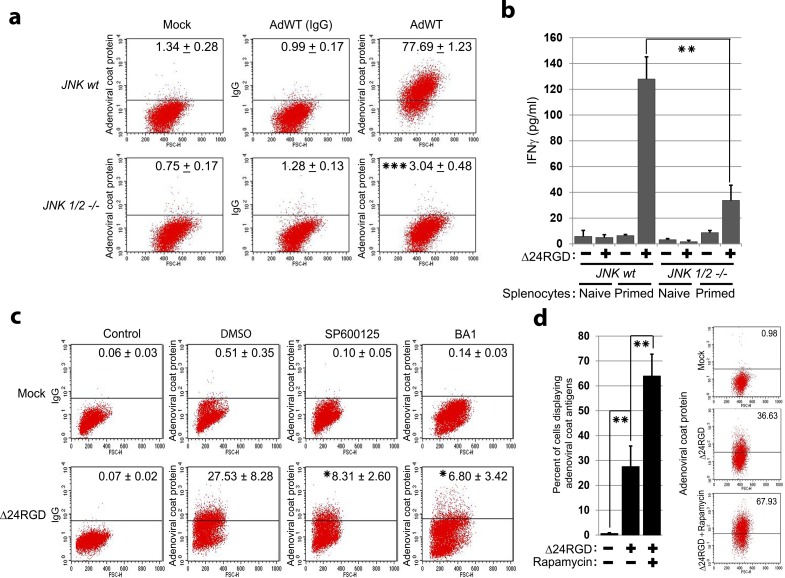
Presentation of adenoviral antigens required JNK expression and activation. (**a**) *JNK wt* and *JNK*1/2-/- MEFs were mock-infected or infected with AdWT (100 MOI) for 48 h and incubated with two combined sets of anti-adenoviral antibodies (a blend of adenoviral coat proteins and adenoviral fiber). They were then incubated with APC-conjugated secondary antibody and analyzed by flow cytometry. IgG isotype was used as a control. Propidium iodide was used to assess cell viability and to analyze live cells. Data represent the percentage of positive cells as mean ± SD. *** *P* < 0.001 (AdWT-infected *JNK*1/2-/- MEFs versus AdWT-infected *JNK wt* MEFs) (unpaired, two-tailed Student *t-test)*. (**b**) Splenocytes were isolated from naive and Δ24RGD-infected C57BL/6 mice and co-cultured with *JNK wt* or *JNK*1/2-/- MEFs that were mock-infected or infected with Δ24RGD adenovirus (100 MOI) for 24 h before co-culture. After 48 h of co-culture, IFN-γ levels (pg/mL) in the conditioned media were assessed by ELISA. Data represent IFN-γ levels (pg/mL) as mean ± SD from three independent co-cultures. ** *P* < 0.01 (unpaired, two-tailed Student *t-test*). (**c**) U87 MG cells were pretreated with DMSO, SP600125 (25 μM), or bafilomycin A1 (BA1, 10 nM) 30 min before infection with Δ24RGD adenovirus (50 MOI) for 48 h. Live cells were incubated with antibodies raised against adenoviral capsid proteins, or IgG isotype as control, and analyzed by flow cytometry. The percentage of APC-positive cells were quantified and represented as mean ± SD (*n* = 3). * *P* < 0.05 (percent of positive cells after treatment with AdWT and SP600125 vs. AdWT and BA1) (unpaired, two-tailed Student *t-test*). (**d**) Significant increase in the presentation of adenoviral antigens in cells infected with Δ24RGD when rapamycin was added to the cultures. U87 MG cells were mock-infected, infected with Δ24RGD, or infected with Δ24RGD and rapamycin (100 nM/48 h) for 48 h and then examined for adenoviral antigens by flow cytometry.

Pathogen-derived antigens may be processed in the autophagolysosomal compartment via autophagy,[10] but the role of autophagy in the presentation of adenoviral epitopes has not yet been determined. With the use of a cocktail of anti-adenoviral protein antibodies, we screened infected cells for the presence of adenovirus-derived peptides on the surface of live, nonpermeabilized, infected cells. For these studies, we took advantage of the fact that JNK-null cells are deficient for autophagy. [15, 16] We observed that whereas the majority (>77%) of *JNK wt* MEFs infected with AdWT expressed adenoviral proteins on their surface, as detected by the FACS analyses (Fig 1A), the genetic ablation of *JNK1* and *JNK2* isoforms resulted in a significant decrease in the percentage of cells testing positive for adenoviral proteins. Because T-cell activation, evidenced by the synthesis and secretion of IFN-γ, is the hallmark method for confirming antigen presentation through interactions between epitope-containing MHC molecules and the T-cell receptor,[26] we substantiated the immune relevance of our data by quantifying the secretion of IFN-γ by splenocytes from uninfected mice (naïve) or adenovirus-treated mice (primed) in co-culture with *JNK wt* or *JNK1/2*-/- MEFs infected with adenovirus. As expected, co-cultures of naïve splenocytes with adenovirus-infected *JNK wt*, or *JNK1/2*-/- cells did not elicit significant secretion of IFN-γ. However, wild-type MEFs infected with adenovirus prompted IFN-γ production when co-cultured with primed splenocytes (Fig 1B). In contrast, cultures of autophagy-deficient *JNK1/2*-/- MEFs with adenovirus-primed splenocytes contained significantly lower secreted IFN-γ levels (Fig 1B). These data showed that autophagy-impaired cells are deficient for the presentation of adenovirus-derived antigens to the immune system.

Further supporting the role of JNK and autophagy in the processing of adenovirus-derived antigens, we observed that treatment of autophagy-proficient infected cultures with JNK inhibitors (SP600125)[27] or autophagy flux inhibitors (bafilomycin A1[28]) significantly decreased the percentage of infected live cells presenting in their surface adenoviral proteins in the FACS analyses (Fig 1C). In agreement with these data, the opposite experiment showed that pretreatment of infected cells with the autophagy-inducer rapamycin[29] resulted in an increase in the percentage of cells that tested positive for adenoviral epitopes in the FACS screening (Fig 1D). Combined, all these observations strongly suggested that autophagy might play a role in the presentation of pathogen-derived antigens in adenovirus-infected cells.

### MHC class II is required for adenoviral antigens

**Fig 2:**
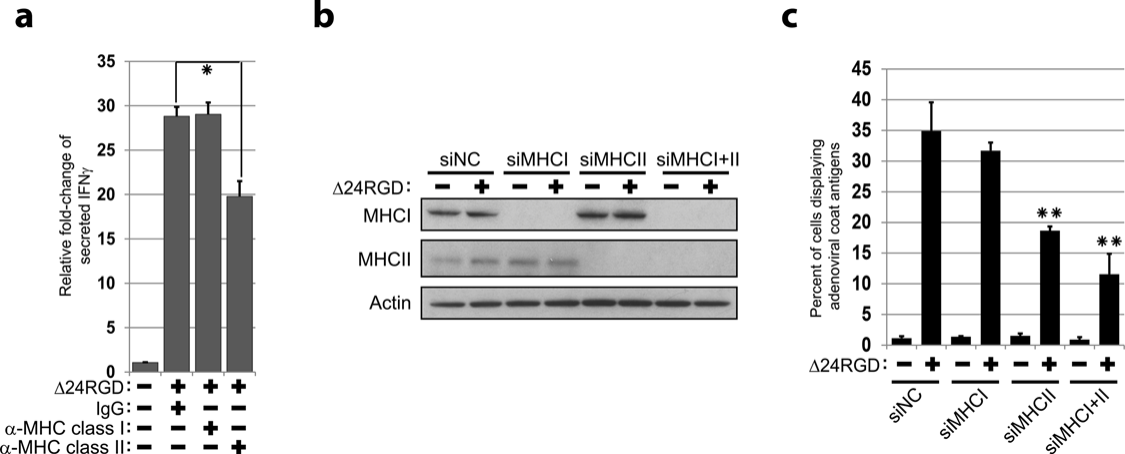
Autophagy is associated with viral antigen presentation. (**a**) Mock- or Δ24RGD-infected wild-type MEFs were pre-incubated with antibodies against MHC class I, MCH class II, or IgG isotope and then co-cultured with splenocytes obtained from Δ24RGD-infected mice. The fold-change of IFN-γ levels (pg/mL) in the co-culture media is displayed relative to control and shown as mean ± SD. ** *P* < 0.01 (unpaired, two-tailed Student *t-test*). (**b**) Wild-type MEF cells were transfected with a pool of siNC, siMHCI, siMHCII, or combined equal amounts of siMHC I and siMHC II for 48 h and then infected with the Δ24RGD adenovirus at an MOI of 10 for an additional 48 h. Whole-cell lysates were analyzed by using anti-MHC class I and anti-MHC class II antibodies. The effect of each siRNA treatment on the protein level of MHC molecules is illustrated. (**c**) Wild-type MEFs were transfected with a pool of siNC, siMHCI, siMHCII, or combined equal amounts of siMHCI and siMHCII for 48 hours, and then infected with the Δ24RGD adenovirus at an MOI of 10 for an additional 48 h. Then, cells were stained for detection of adenoviral antigens. Propidium iodide was used to assess cell viability. Data are shown as percentage of positive cells (mean ± SD). The decrease in the percent of positive adenovirus-infected, siMHCII-transfected cells compared with adenovirus-infected, siNC-transfected cells was statistically significant. ** *P* < 0.01 (unpaired, two-tailed Student’s *t-test)*.

Autophagy is involved in the processing of peptides for presentation mainly via MHC class II molecules,[10, 11] thus we decided to determine whether antibody-based blockade of MHC class II prevented the recognition of infected cells by the adenovirus-primed splenocytes. To this end, we quantified the IFN-γ production in co-cultures of adenovirus-primed splenocytes with virus-infected MEFs pre-incubated with blocking antibodies for MHC class I or II. We observed that cells treated with antibodies against MHC class II proteins showed significant inhibition of IFN-γ production compared with cells treated with antibodies against MHC class I proteins or IgG-treated controls (Fig 2A). To confirm these data we challenged the presentation of adenovirus antigens by using specific siMHC class II (Fig 2B). We observed that down-modulation of MHC class II mRNA resulted in the decrease in the number of cells presenting adenoviral epitopes (Fig 2C). Indicating the predominant role of the MHC class II in the process, showed that the down-modulation of MHC class I had much less effect on the presentation of adenovirus-derived antigens than did the down-regulation of MHC class II. These data indicate that adenovirus-derived antigens are predominantly presented via MHC class II, the predominant pathway of epitope presentation for proteins processed via autophagy.[10, 11]

### Adenoviral structural proteins interact with p62 and are degraded in the autolysosome

**Fig 3:**
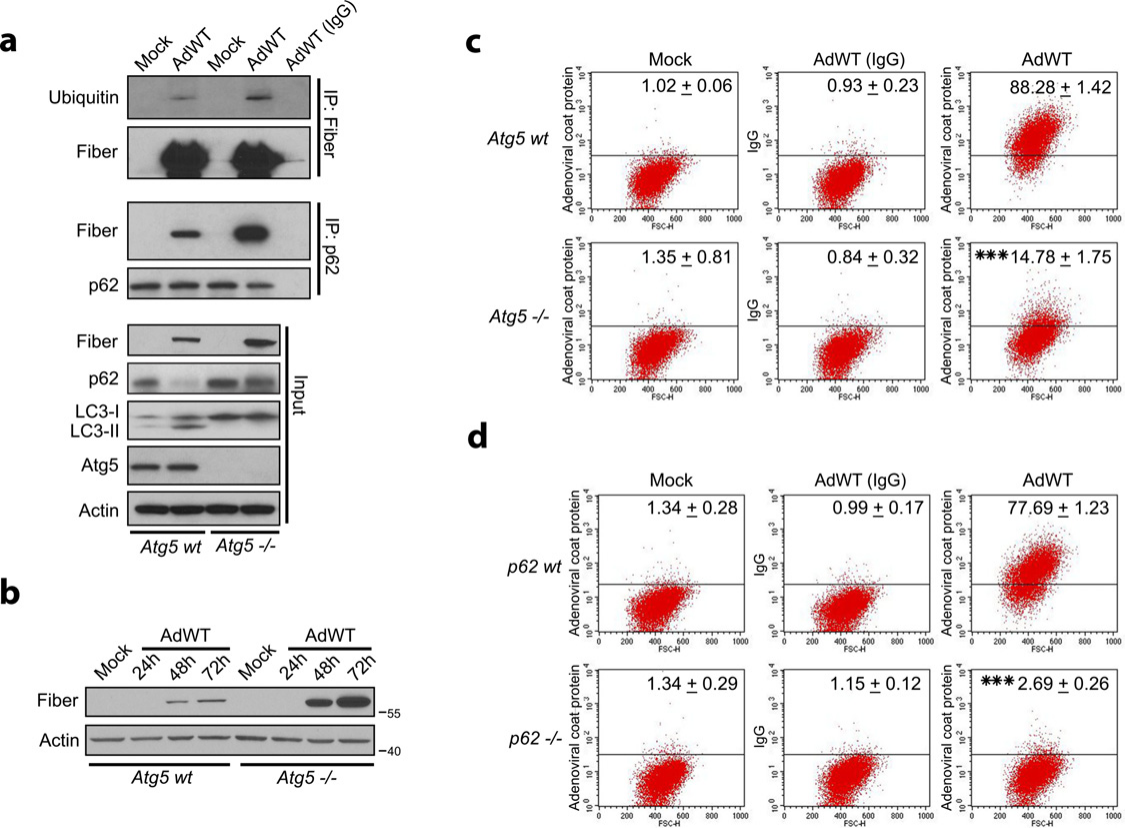
Autophagy is involved in the processing of adenovirus-derived epitopes. (**a**) Cell lysates from *Atg5wt* or *Atg5-/-* MEFs infected with AdWT (50 MOI) were analyzed for fiber/ubiquitin and fiber/p62 protein complexes. LC3-I to LC3-II conversion and p62 expression levels were analyzed in an input sample (5%). Actin is shown as a loading control. (**b**) Cell lysates from *Atg5wt* and *Atg5*-/- MEFs were mock-infected or infected with AdWT (50 MOI) for the indicated times and analyzed for adenoviral fiber expression. Actin was used a loading control. (**c**) *Atg5wt* and *Atg5-/-* MEFs were mock-infected or infected with AdWT adenovirus (50 MOI) for 48 h and then stained with antibodies to adenoviral coat proteins, incubated with APC-conjugated secondary antibody, and analyzed by flow cytometry. IgG isotype was used as a control. Propidium iodide was used to assess cell viability. Data are shown as percentage of APC-positive cells (mean ± SD) of three independent experiments. *** *P* < 0.001 (percentage of APC-positive in AdWT-infected *Atg5wt* cells vs. AdWT-infected *Atg5*-/- cells) (unpaired, two-tailed Student *t-test*). (**d**) *p62wt* and *p62-/-* MEFs were infected with AdWT adenovirus (100 MOI) for 48 h and then stained with antibodies against adenoviral coat proteins, incubated with APC-conjugated secondary antibodies, and analyzed by flow cytometry. IgG isotype was used as a control. Propidium iodide was used to assess cell viability. Data are shown as percentage of APC-positive cells (mean ± SD) of three experiments. *** *P* < 0.001 (percent of APC-positive cells in AdWT-infected *p62*-/- vs. AdWT-infected *p62wt* cells) (unpaired, two-tailed Student *t-test*).

Next we sought to examine whether structural adenoviral proteins were degraded in the autophagolysosomes. Because the p62 chaperone protein binds to ubiquitinated proteins for their sequestration and degradation in autophagolysosomes,[30] we analyzed the potential interaction between p62 and fiber protein performing co-immunoprecipitaton experiments. We showed that the adenoviral fiber protein was ubiquitinated during adenovirus infection and interacted with p62 (Fig 3A). Consistent with a role of the autophagolysosome in the degradation of adenoviral fiber protein, the interactions of p62 and fiber protein were more evident in *Atg5*-/- MEFs, which are deficient for adenovirus-induced autophagy [7] (Fig 3A). In fact, detailed examination of fiber protein levels at several time points after adenoviral infection revealed remarkably higher levels of these proteins in autophagy-deficient *Atg5-/-* MEFs cells compared with wild-type *Atg5* MEFs (*Atg5 wt*) infected with equal amounts of wild-type adenoviruses (Fig 3B). Moreover, we also observed that after adenoviral infection there was a significant decrease in the percentage of cells that presented adenoviral proteins in the *Atg5-/-* MEFs cultures compared with *Atg5wt* MEFs (Fig 3C). As expected, similar results were observed with *p62*-/- MEFs infected with adenovirus, confirming that lack of *p62* expression resulted in a significant reduction of the percentage of adenoviral coat-positive cells with respect to adenovirus-infected wild-type *p62* MEFs (Fig 3D), probably due to the deficient p62-mediated transport of adenoviral proteins to the autophagosome. These data further suggested that adenoviral proteins were degraded in the autophagolysosomes during active autophagic flux resulting in presentation of adenovirus-derived epitopes at the host cell surface.

### Generation of Δ24FvIII adenovirus

**Fig 4:**
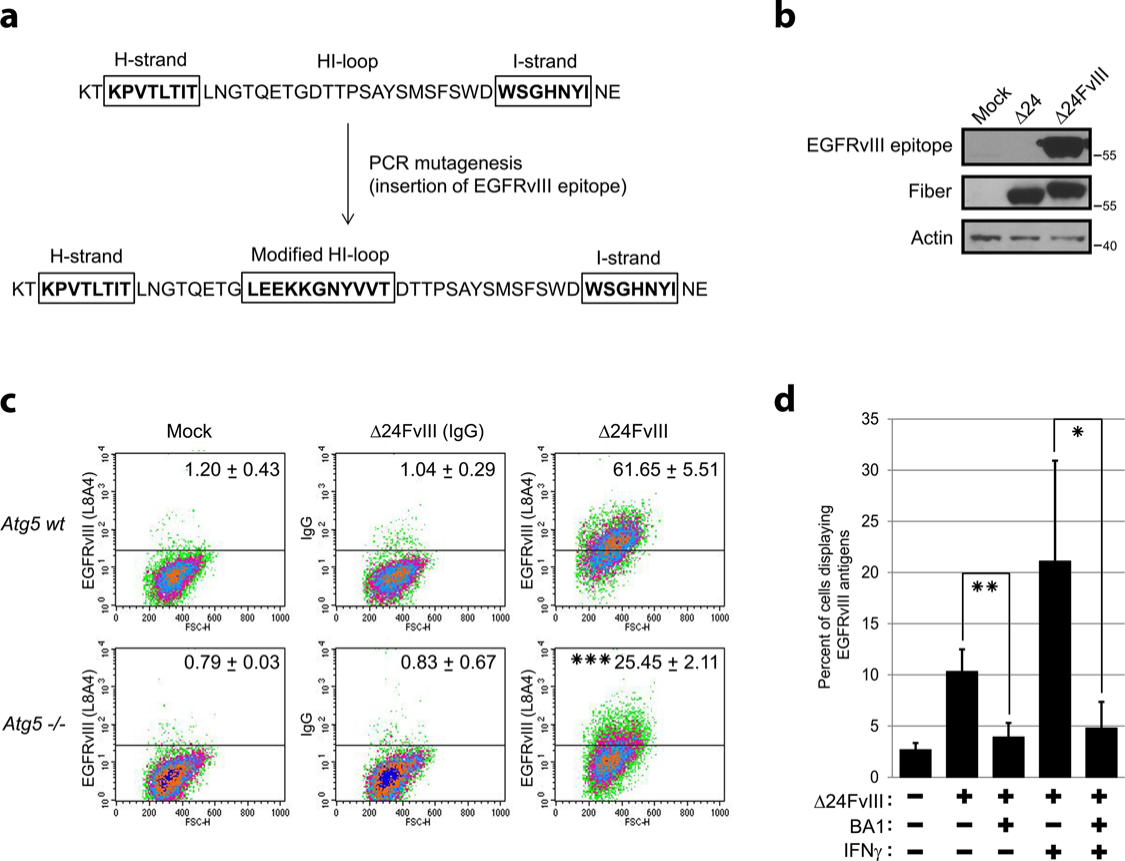
Infection with Δ24FvIII adenovirus induces EGFRvIII epitope expression and presentation. (**a**) Schematic representation of the generation of the vIII chimeric fiber. PCR-based mutagenesis was used to insert the sequence of the LEEKKGNYVVT epitope in the hypervariable region of the HI loop. A unique EcoRV restriction site was incorporated to allow the insertion of the ectopic sequence between glycine-543 and aspartic acid-544. (**b**) Expression of the chimeric fiber encompassing the LEEKKGNYVVT peptide was assessed in A549 cells infected with Δ24FvIII (40 MOI). Protein lysates were subjected to Western blot analysis 72 h after infection using both anti-fiber and L8A4 anti-LEEKKGNYVVT antibodies. (**c**) Live wild-type *Atg5* and *Atg5*-/- MEFs were infected with Δ24FvIII at an MOI 150 for 48 h and then stained with L8A4, followed by FITC-conjugated secondary antibodies for flow cytometry analyses. Ethidium homodimer-1 staining was used to exclude dead cells. Percentages of FITC-positive live cells are indicated at the top right corner of each graph. The decrease in the number of positive cells in Δ24FvIII-infected *Atg5*-/- compared with Δ24FvIII-infected wild-type *Atg5* cells was statistically significant. Data are shown as the mean ± SD of three experiments. *** *P* < 0.001 (unpaired, two-tailed Student *t-test*). (**d**) HeLa cells were infected with Δ24FvIII at an MOI of 40. IFN-γ (300 units/mL) or/and bafilomycin A1 (BA1; 100 nM) were added to the media 6 h or 24 h after infection, respectively. Live cells were stained with L8A4 48 h after infection and then incubated with FITC-conjugated secondary antibodies to visualize positive cells with a flow cytometer. Ethidium homodimer-1 staining was used to exclude dead cells. Graph represents mean values of three experiments ± SD. * *P* < 0.05; ** *P* < 0.01 (unpaired, two-tailed Student *t-test*).

Modification of adenoviral capsids by insertion of antigens sequences is a promising technology for the development of vaccines and anti-cancer vaccines,[31] and therefore we sought to determine whether autophagy was the main mechanism for the processing of ectopic cancer-specific epitopes encoded by adenoviral fibers. We then generated an experimental model that would allow us to determine whether a specific sequence in the adenoviral fiber could be detected in adenovirus-infected cells. To this end, we designed and constructed a chimeric adenovirus fiber encompassing the sequence of a well-characterized human epitope (Fig 4A). The resulting construct was termed Δ24FvIII, and used the Δ24 tumor-selective backbone adenovirus[32] and encoded the epitope LEEKKGNYVVT inserted into the HI-loop of the adenovirus fiber sequence. This peptide is derived from the junctional sequence of the truncated protein generated in the mutant variant III of the mutant EGFR and has been previously shown to be highly immunogenic[21] (Fig 4A).

### Cells infected with F_21" \o "Humphrey, 1990 #149" thEGFRvIII-derived cancer-specific epitope

Using a highly specific antibody generated against the EGFRvIII peptide[21], we documented that the expression of the chimeric fiber was easily detected in Δ24FvIII-infected cells (Fig 4B). We hypothesized that the ectopic sequence of the human epitope inserted into the adenoviral fiber was processed via autophagy and presented in the surface of infected cells. Therefore, after wild type and autophagy-deficient *Atg5*-/- MEFs were infected with Δ24FvIII, the surfaces of live cells were examined for the presence of LEEKKGNYVVT with the use of the specific antibody[21] and FACS analyses. We showed that a high percentage of wild-type *Atg5* MEFs were positively identified by the anti-LEEKKGNYVVT antibody (Fig 4C). However, the percentage of positive cells was significantly decreased in autophagy-deficient *Atg5*-/- MEFs infected with Δ24FvIII adenovirus (Fig 4C). Similar data were obtained when HeLa cells were infected with Δ24FvIII adenovirus and the autophagy flux was blocked with bafilomycin A1. Thus, the percentage of HeLa cells presenting the LEEKKGNYVVT peptide decreased dramatically when the infected cultures were treated with bafilomycin A1 (Fig 4D). Strongly suggesting the specific activation of the antigen-processing machinery during infection, the presentation of the LEEKKGNYVVT peptide increased after the addition of IFN-γ, which transcriptionally activate MHC,[33] to cultures infected with Δ24FvIII adenovirus (Fig 4D). Finally, further demonstrating the role of autophagy in the processing of LEEKKGNYVVT, bafilomycin A1 pretreatment induced a blocking effect that antagonized and significantly decreased the positive modulation of antigen presentation mediated by IFN-γ (Fig 4D).

## Discussion

Our data provide evidence for the first time that inhibition of autophagy in adenovirus-infected cells prevents efficient presentation of adenovirus-derived and fiber-incorporated cancer-specific epitopes. We have also demonstrated that adenoviral proteins interact with the autophagy cargo-receptor p62 protein, strongly suggesting that p62 may carry adenoviral capsid proteins to the autophagosome. Interestingly, p62 has been proposed to work as a potential sensor for virus infection and the link between virus proteins and autophagy.[34] The fact that p62 binds adenoviral fiber strongly suggested that autophagy was involved in adenoviral protein degradation. In fact, we had presented in a previous work the preliminary observation that adenovirus-infected cells deficient for autophagy showed higher levels of fiber protein, and that modulation of autophagy did not decrease viral replication.[7, 16] Autophagy-mediated degradation of adenoviral proteins strongly suggests that autophagy participates in the processing of adenovirus-derived antigens,[9–11] and therefore that autophagy may also be the mechanism for the presentation of capsid-integrated ectopic antigens.[31] In agreement with this, and suggesting that autophagy may play a broad role in the processing of antigens by other viruses, Paludan and colleagues reported that endogenous Epstein-Barr virus nuclear antigen 1 (EBNA1) was found to gain access to the MHC class II pathway by autophagy.[10] Thus, upon inhibition of lysosomal acidification, EBNA1 antigen, similar to what we observed with adenoviral fiber, slowly accumulated in cytosolic autophagosomes. In addition, inhibition of autophagy decreased recognition of EBNA1 transduced cells by EBNA1-specific CD4+ T cell clones.[10]

Our data suggest that adenoviral antigens are processed through autophagy and presented predominantly via MHC class II.[10] It has been reported that MHC class II presentation of antigens leads first to the activation of CD4^+^ cells, and then to stimulation of CD8^+^ cytotoxic T cells, that will ultimately execute the anti-cancer activity [35]. Our data are also in agreement with the main function of autophagy in antigen processing[9–11] and with previous reports suggesting that autophagy may be the main mechanism for the generation of virus-derived antigens.[9–11] In the particular scenario of adenoviral infection, another circumstance promoting a dominant MHC class II antigen presentation is the induction and maintenance of a highly productive autophagic flux in the infected cells.[10, 36] Furthermore, the presentation of the epitope via MHC class I may likely be attenuated due to the down-regulation of MHC class I complexes induced by the E3-19k adenoviral protein.[37]

The capsid of oncolytic adenoviruses, such as Delta-24, can be modified for many purposes including the transfer and expression of cancer-specific epitopes that will redirect the strong immune response elicited by the adenovirus infection to the infected, and potentially to uninfected, cancer cells.[25, 31] Importantly, because Delta-24 has been previously modified to include an RGD-4C sequence in the fiber knob, this tropism-enhanced construct not only can efficiently infect CAR-deficient cancer cells[1] but also should demonstrate an enhanced capability to infect dendritic cells.[38] Our data indicate that these cancer-vaccine strategies can be combined with autophagy-inducers such as rapamycin. This combination will probably produce a synergistic antitumor effect because of the direct effect of the drug and the viral oncolysis[39, 40] and because of the enhancement of autophagy, which should lead to increased virus-mediated cell lysis,[7] and perhaps more relevant for the development of a permanent therapeutic effect, to the presentation during the adenoviral infection of cancer epitopes incorporated in the adenoviral fiber.

In summary our data show that induction of autophagy during adenoviral infection is required for the efficient presentation of viral antigens and this concept should be considered for the development and testing of adenovirus based cancer vaccines.
